# Incidence, time to occurrence and predictors of peripheral intravenous cannula-related complications among neonates and infants in Northwest Ethiopia: an institutional-based prospective study

**DOI:** 10.1186/s12912-022-01164-x

**Published:** 2023-01-11

**Authors:** Nega Dagnew Baye, Assefa Agegnehu Teshome, Atalo Agimas Ayenew, Tadeg Jemere Amare, Anmut Tilahun Mulu, Endeshaw Chekol Abebe, Gebrehiwot Ayalew Tiruneh, Teklie Mengie Ayele, Zelalem Tilahun Muche, Awgichew Behaile Teklemariam, Biruk Demissie Melese, Melaku Mekonnen Agidew, Mohammed Abdu Seid

**Affiliations:** 1 Department of Biomedical Sciences, College of Health Sciences, Debre Tabor University, Debre Tabor, Ethiopia; 2 Department of Clinical Midwifery, College of Health Sciences, Debre Tabor University, Debre Tabor, Ethiopia; 3 Department of Pharmacy, College of Health Sciences, Debre Tabor University, Debre Tabor, Ethiopia; 4 Department of Environmental Health, College of Health Sciences, Debre Tabor University, Debre Tabor, Ethiopia

**Keywords:** Peripheral intravenous cannula, Peripheral intravenous cannula complications, Incidence, Infants, Infiltration, Phlebitis

## Abstract

**Background:**

Peripheral intravenous cannulas (PIVC) are venous access devices commonly used for the administration of intravenous fluids, drugs, blood products, and parenteral nutrition. Despite its frequent use, it has complications that can seriously threaten patient safety, prolong hospital stays, and increases medical care costs. PIVC complications are associated with increased morbidity and reinsertion attempts are painful and anxiety-provoking for children and their parents. Therefore, this study was aimed to assess the incidence, time to occurrence and identify predictors for PIVC complications among infants admitted to Debre Tabor Comprehensive Specialized Hospital (DTCSH), Northwest Ethiopia.

**Methods and setting:**

An institutional-based prospective cohort study was conducted on 358 infants admitted to a neonatal intensive care unit and pediatric ward, DTCSH from January 1 to April 30, 2022. A systematic sampling technique was employed.

**Results:**

The incidence rate of PIVC complication was 11.6 per 1000 person-hours observation. PIVC complication was observed in 56.4% (202) of PIVCs, of which infiltration (42.1%) was the most common complication followed by phlebitis (29.7%). The median time to complication was 46 h. Anatomical insertion site (AHR = 2.85, 95%CI: 1.63–6.27)**,** admission unit (AHR = 1.88, 95%CI: 1.07–4.02), sickness (AHR = 0.24, 95% CI: 1.31–4.66), medication type (AHR = 2.04, 95%CI: 1.13–3.66), blood transfusion (AHR = 0.79, 95%CI: 0.02–0.99), clinical experience (AHR = 0.52, CI:0.26–0.84), and flushing (AHR = 0.71, 95%CI: 0.34–0.98) were potential predictors of PIVC complication.

**Conclusion:**

Knowing the predictor factors helps clinicians to provide effective care and to detect complications early.

## Background

A peripheral intravenous cannula (PIVC) is a short catheter inserted into the vein in the peripheral areas of the patient [1]. PIVC insertion is one of the most frequently performed invasive procedures in hospitalized neonates and infants. It is estimated that 30% to 80% of admitted patients undergo this procedure during hospitalization [2]. It provides venous access for the administration of intravenous fluids, drugs, blood products, parenteral nutrition, and sampling of blood [3].

Despite its benefits and frequent use, intravenous cannulation has complications that can seriously threaten patient safety such as clotting, occlusion, leakage, infiltration, extravasation, phlebitis, and infection [4]. Furthermore, it is the main source of procedure-related pain in hospitalized patients [5].

In neonates and infants, PIVC complications are associated with increased morbidity [6]. PIVC failure due to complications and reinsertion attempts are painful and anxiety-provoking for neonates and infants and their parents. It interferes with the most important intravenous therapy, resulting in longer hospital stays and higher additional medical care costs, including the expense of PIVC and nursing time [7, 8].

Ill neonates and infants are at an increased risk of PIVC-related complications. The risk of a PIVC-related complication in neonates and infants is reported as up to 75%, of which extravasation/infiltration rate was high, having an incidence of around 65% [4, 9–11]. Occlusion is also a common cause of premature loss of catheter function [12]. This can be attributed to their immature skin structure, immature immune system, flexible subcutaneous tissue, and smaller, fragile veins [9, 13]. Globally, most peripheral intravenous cannulas are inserted into the hand [8].

In neonatal intensive care units (NICU), local complications occur in up to 97% of PIVCs, and the cannulas have a median lifespan of 23 to 40 h [4, 10, 14]. For instance, in Jordan, the average life span of PIVCs was 18.45 h and the incidence of phlebitis started increasing, from 3.7% to 21.2%, after 24 h and reached 27.5% after 48 h [15].

Many factors, including infant age, weight, corticosteroid use, flushing, cannula size, anatomical site of insertion, type of infusion, and infection, influenced the development of PIVC complications [9, 10, 16, 17].

The incidence of PIVC complications is still higher and remains an issue of public health concern. There is certainly a pressing need for credible research and forwarding suggestions in this area. To the best of our knowledge, there is no published scientific research describing the incidence and time to complication of PIVC and its predictors among neonates and infants in Ethiopia. Therefore, this study was primarily aimed to assess the incidence, types, and time to occurrence of PIVC complications and to identify predictors for PIVC complications among infants admitted to Debre Tabor Comprehensive Specialized Hospital, Northwest Ethiopia. Our findings could provide directions for the prevention of PIVC complications, improvement of patient outcomes, and reduction of medical care costs.

## Methods

### Study design and setting

This was an institutional-based prospective cohort study conducted among 358 neonates and infants admitted to Debre Tabor Comprehensive Specialized Hospital, NICU and pediatric ward from January 1 to April 30, 2022. The hospital is located in Debre Tabor town which is the zonal center of South Gondar Zone, Amhara region, Northwest Ethiopia. The town is located 667 km from Addis Ababa and 98 km away from Bahir Dar. The hospital serves around 2.2 million population across the region and provides a broad range of medical services for all age groups. The pediatric ward had an average monthly admission of 55 infants, and the NICU was also fairly equipped with an average of 126 neonatal admissions per month and provides a tertiary level of care.

### Population and eligibility criteria

The study population was all neonates and infants admitted to NICU and pediatric ward in DTCSH who required intravenous therapy during the study period.

Eligible participants were all neonates and infants for whom consent was given by their mothers/caregivers and who required intravenous therapy. However, those who were admitted for observation only, referred from another department with preexisting PIVC, and those who had PIVCs inserted for diagnostic test (for temporary use) were excluded from the study.

### Study variables

#### Dependent variable

Occurrence of PIVC complication.

#### Independent variables

Included infant's demographic and clinical-related variables (sex, weight, age, admission unit, indication for cannula securement, admission diagnosis, skin integrity, and sickness), intravenous cannula-related variables (cannula size, anatomical site of insertion, and venous condition), health professional and environmental factors (intravenous insertor (profession), educational level, clinical experience, number of attempts to insert, flushing or irrigation, immobilization material, type of dressing).

### Sample size estimation and sampling technique

The sample size was computed using a single population proportion formula by considering the incidence of PIVC complications as 63.15% [10], 95% confidence interval (CI), and 5% margin of error. The final sample size was 358. The study participants were selected using a systematic random sampling technique. The total sample size was proportionally allocated for the NICU and pediatric ward. Using the formula, by considering N (total number of neonates and infants admitted during the data collection period) which was 724, n (required minimum sample size was 358) and K- interval (K = N/n =  > 724/358 = 2). The first participant was selected through lottery method by drawing a number from 1 and 2, the number two was selected and continuing with every second neonate or infant until the final sample size was reached. If neonate or infant does not satisfy the inclusion criteria, then the next participant was selected.

### Operational definitions

Time to PIVC complication: is the time in hours from the insertion of PIVC to the identification of complication before 96 h.

Event: The development of PIVC complications before 96 h.

Censored: Infants did not experience the event of interest before 96 h.

Phlebitis: was defined as the presence of two or more signs of pain, tenderness, warmth, erythema, swelling, or a palpable cord with or without purulent drainage from the catheter insertion site [16].

Infiltration/extravasation: was defined as the unintended permeation of intravenous fluid into the surrounding interstitial compartment, causing swelling of the tissue around the site of the cannula, where infiltration is the infusion of non-vesicant fluids or medication and extravasation is the infusion of vesicants into surrounding tissues [9, 17].

Occlusion: is the resistance to the passage of an intravenous solution or medication.

### Data collection procedures and tools

The data were collected using a properly designed, pretested, well-structured observational checklist and structured questionnaire, which were adapted and prepared after reviewing relevant literatures from previously conducted studies. The observational checklist and structured questionnaire had demographic variables of infants (sex, age, weight), clinical data of the infants (type of diagnosis, sickness, and admission unit), cannula-related characteristics (size of device, the date and time of cannulation, the date and time of removal of the PIVC, type and time to complication, number of attempts, anatomical site of cannula insertion, indication for cannulation (intravenous fluids, medications, blood transfusion)), and health care and environmental factors (type of fluid, infusion type, profession, education, and clinical experience of cannula inserter, immobilization material used, and medication type). A balance beam neonate scale and thermometer were used to measure the weight and body temperature of the participants, respectively. All participants were followed up for 96 h for PIVC during the hospital stay and the changes were recorded. Data collectors assessed the PIVC site every hour for the development of complication. The data were collected from the first cannula insertion time until its complication and removal. The data were collected by five medical doctors who work in the hospital.

### Follow up of participants

Recruitment of infants took place at NICU and pediatric ward immediately at admission. Firstly, data collectors asked infants mother/caregiver for consent and then enrolled. Finally, follow-up of infants was carried out for 96 h until the development PIVC complication.

### Statistical analysis

Statistical analysis was conducted using STATA 14 statistical software. Continuous variables were summarized as means with standard deviations. Categorical variables were expressed as count and percentage (%) and presented in tables and charts. Kaplan Meier failure curve was used to estimate the probability of complication and the incidence rate was calculated. The equality of survivor functions was assessed by a log-rank test. First, bivariable Cox proportional hazard regression was performed. Then, variables with a *p*-value < 0.25 were included in a multivariable Cox proportional hazard regression analysis to find the predictors of PIVC complication. The Schoenfeld residuals proportional hazard assumption test for each variable and the overall global test were done. The Schoenfeld residuals test confirmed that the assumption was met where the *p*-value was > 0.05 for each variable as well as the overall global test (*p*-value = 0.38). Finally, it can be concluded that the final model fits the data well. The strength of the association was measured using adjusted hazard ratios (AHR) with a 95% confidence interval and *p*-values of < 0.05.

## Results

In this study, 358 admitted neonates and infants were included, of which 242 (67.6%) of them were males. The age ranges from 0–12 months with a mean of 3.87 ± 2.4 months. The weight ranged from 1 to 14 kg, with a mean of 3.9 ± 2.5 kg. Two hundred forty-nine (69.6%) neonates were admitted to NICU. The commonest disease cause for admissions was respiratory diseases (47.2%), followed by gastrointestinal problems (21.5%) (Table 1).

**Table 1:** Socio-demographic and clinical characteristics of neonates and infants admitted to DTCSH, Northwest Ethiopia, 2022

Variables		Frequency (%)
**Sex**	Male	242(67.6%)
	Female	116(32.4%)
**Weight**	≤ 1.49 kg	13(3.6%)
	1.5–2.49	82(22.9%)
	2.5–3.49	130(36.3%)
	≥ 3.5	133(37.2%)
**Temperature**	< 36.5	148(41.3%)
	36.5–37.5	86(24%)
	> 37.5	124(34.6%)
**Pediatric unit**	NICU	249(69.6%)
	Pediatric ward	109(30.4%)
**Admission Diagnosis**	Gastrointestinal problems	77(21.5%)
	Respiratory problems	169(47.2%)
	Hematologic problems	30(8.4%)
	Metabolic disorders	9(2.5%)
	Others	73(20.4%)
**Sickness**	Acute	323(90.2%)
	Chronic	35(9.8%)

For all infants, PIVC insertion was accomplished by 24-gauge cannulas. Majority (63.7%) of cannulas were inserted into veins on the dorsum of the hand, followed by 66 (18.4%) punctures on the dorsum of the feet. While, in 10.1% PIVC was inserted on anti-cubital fossa. About 92.5% of peripheral intravenous cannulas were attached to the insertion site by sterile zinc oxide adhesive plaster. More than half of (328, 91.6%) PIVCs were inserted into veins that were visible.

In about 254 (70.9%) cannulas, insertion was accomplished in 2 or more attempts. About 219 (61.2%) nurses who inserted the cannula had 2–3 years of clinical experience, and 152 (42.5%) were neonatal nurses. About 335 (93.6%) peripheral intravenous cannulas were placed for administration of medication. Of which, antibiotics were the most commonly administered medications accounting for 88.4% of PIVCs. About 84(23.5%) peripheral intravenous cannulas were placed for fluid administration, 65 (77.4%) children were resuscitated with maintenance fluid, and 55 (65.5%) were infused continuously. Flushing was done in majority of PIVCs (289, 80.7%).

During the follow-up period, 202 (56.4%) new PIVC complication cases were observed, of which infiltration (42.1%) was the most frequently encountered complication followed by phlebitis (29.7%). The median time to PIVC-related complication in infants was 46 h (95% CI = 43, 48), and the mean time to complication was 48.7 h (95% CI = 45.9, 51.5). Infants were followed for a minimum of four hours to a maximum of ninety-six hours, which provides a total of 17,442 person-hours of observation and a 0.01158124 incidence rate. The Incidence rate of PIVC complication was 11.6 per 1000 Person -hours of observation. Most cannulas (40%) develop complications between 24–48 h. The sites with more complications were the lower limb (71.2%) followed by the cubital fossa (61.1%).

### Log-rank test result comparison on different categorical variables

Log-rank test was performed to test the equality of complication curves of different categorical explanatory variables. The test statistics showed that there is a significant difference in the probability of PIVC complication occurrence at any time point between groups of the following categorical variables: infection (log-rank *p*-value = 0.003), admission unit (log-rank *p*-value = 0.04), anatomical site of cannula insertion (log-rank *p*-value = 0.038), flushing (log-rank *p*-value = 0.048), medication type (log-rank *p*-value = 0.004), blood transfusion (log-rank *p*-value = 0.003), clinical experience (log-rank *p*-value = 0.004).

### Predictors of PIVC complication in neonates and infants

Variables with a *p*-value < 0.25 on bivariable Cox proportional hazard regression were entered into a multivariable cox proportional hazard regression to control possible confounders and to identify statistically significant predictors of PIVC complications. Accordingly, in multivariable Cox proportional hazard regression, age group, cannula insertion site**,** admission unit, sickness, medication type, blood transfusion, clinical experience, and flushing remains significant predictors of PIVC complication.

The hazard of developing PIVC complications for those infants with acute infection was 2.4 times higher (AHR = 2.4, 95% CI: 1.31–4.66) when compared to those who had a chronic illness. Flushing of the cannula after securement decreases the risk of PIVC complications by 29% (AHR = 0.71, 95%CI: 0.34–0.98) as compared to intravenous cannulas which were not irrigated (Table 2).

**Table 2:** Cox-proportional hazards regression analysis to determine the predictors of PIVC complication among infants admitted in DTCSH, Northwest Ethiopia, 2022

Variables	Out come		AHR (95%CI)	*P*-value
	**Complication (%)**	**Censored (%)**		
**Sex**				
** Female**	64 (55.2%)	52 (44.8%)	1	
** Male**	138 (57%)	104 (43%)	1.68 (0.77–3.65)	0.19
**Admission unit**				
** Pediatric ward**	62 (56.9%)	47 (43.1%)	1	
** NICU**	140 (56.2%)	109 (43.8%)	1.88 (1.07–4.02)	0.04^*^
**Anatomical site of cannula insertion**				
** Scalp**	15 (53.6%)	13 (46.4%)	1	
** Dorsum of hand**	118 (51.8%)	110 (48.2%)	1.22 (0.63–2.45)	0.07
** Cubital fossa**	22 (61.1%)	14 (38.9%)	2.03 (1.13–6.08)	0.003^*^
** Leg**	47 (71.2%)	19 (28.8%)	2.85 (1.63–6.27)	0.0001^*^
**Admission diagnosis**				
** Gastrointestinal problem**	37 (48.1%)	40 (51.9%)	0.70 (0.45–1.09)	0.12
** Respiratory problem**	106 (62.7%)	63 (37.3%)	0.93 (0.65–1.33)	0.71
** Hematologic problem**	14 (46.7%)	16 (53.3%)	0.61 (0.33–1.11)	0.11
** Metabolic problem**	3 (33.3%)	6 (66.7%)	1.06 (0.33–3.42)	0.92
** Others**	42 (57.5%)	31 (42.5%)	1	
**Medication type**				
** Antibiotics**	166 (56.1%)	130 (43.9%)	1	
** Corticosteroid**	11 (47.8%)	12 (52.2%)	2.04 (1.13–3.66)	0.018^*^
** Others**	12 (75%)	4 (25%)	0.77 (0.42–1.43)	0.42
**Residency**				
** Rural**	103 (54.5%)	86 (45.5%)	1	0.787
** Urban**	99 (58.6%)	70 (41.4%)	1.1 (0.51–2.4)	
**Flushing**				
** Yes**	170 (58.8%)	119 (41.2%)	0.71 (0.34–0.98)	0.01^*^
** No**	32 (46.4%)	37 (53.6%)	1	
**Blood transfusion**				
** Yes**	1 (8.3%)	11 (91.7%)	0.79 (0.02–0.99)	0.04^*^
** No**	191 (55.2%)	155 (44.8%)	1	
**Clinical experience**				
** ≥ 12 years**	11 (57.9%)	8 (42.1%)	0.93 (0.25–3.35)	0.76
** 6–11 years**	43 (38.4%)	69 (61.6%)	0.52 (0.26–0.84)	0.04^*^
** 3–5 years**	146 (66.6%)	73 (33.4%)	0.31 (0.11–0.65)	0.82
** ≤ 2 years**	3 (37.5%)	5 (62.5%)	1	
**Sickness**				
** Acute**	189 (58.5%)	134 (41.5%)	2.4 (1.31–4.66)	0.01^*^
** Chronic**	13 (37.1%)	22 (62.9%)	1	

## Discussion

Although PIVC is a commonly performed clinical procedure, it is associated with significant risks and complications. Various studies on pediatric population reported the rate of PIVC complications within the range of 34% to 56% [7]. In this study, there is a high incidence of PIVC complications among admitted neonates and infants, which were observed in more than half of the participants (56.4%), and this result was slightly higher than the report of previous studies [14, 15, 18]. However, it was slightly lower than the result reported in Brazil [10]. In the current study, the most frequently observed complications were infiltration (42.1%) and phlebitis (29.7%). A similar finding was noted in previous studies [10, 17, 19]. Furthermore, a study in Janeiro, Brazil reported that infiltration was the primary reason for withdrawal of peripheral venous access [20]. However, a lower incidence was reported in Tunisia [14]. On the other hand, studies conducted in Jordan and Ambala, Haryana reported a higher incidence of intravenous cannula-associated phlebitis [16, 21]. Regarding the rates of occlusion, the present study found a comparable result with the reports from previous studies [10, 20].

The most common anatomical site of cannula insertion was on the dorsum of the hand which is supported by various studies [8, 14, 17, 18]. However, in Jerusalem, Israel the common anatomic site of insertion was the antecubital fossa [15].

In the current study, anatomical site of cannula insertion**,** admission unit, sickness, medication type, blood transfusion, clinical experience, and flushing remain potential predictors of PIVC complications among admitted infants with PIVCs. The study found that the risk of developing PIVC complications was higher in those infants with acute infection. This is consistent with studies from Brazil [10] and Turkey [9] where neonates with acute infection were more likely to develop complications. Neonates admitted to NICU had a higher risk of developing complications, which is inline with a study conducted in Turkey [9] where immature newborns were more likely to have complications. This might be due to neonates had comparatively immature skin structure, flexible subcutaneous tissue, small veins, and poor venous integrity. However, a study in Mexico revealed that the risk of complications was higher in older pediatric age group of patients [22].

In the current study, cannulation on the lower limb showed the highest complication rate which is supported by previous studies [15–17]. The use of normal saline for flushing maintains patency and avoids PIVC complications which is inline with the finding of the present study [23, 24]. In the current study, the clinical experience of a nurse who insert the cannula had a significant effect on the incidence of PIVC complications which is comparable with the study in Mekelle [25].

## Conclusion

In the current study, the incidence of PIVC-related complications was high with a large proportion of PIVC complications occurring in the form of infiltration and phlebitis. Anatomical site of cannula insertion**,** admission unit, sickness, medication type, blood transfusion, clinical experience of the inserter, and flushing were the predictor factors associated with PIVC complications.

Therefore, all infants with PIVC should be screened for complications. Knowing the predictor factors of PIVC complications helps clinicians to provide effective care and to detect complications early.

## Data Availability

The datasets generated and/or analyzed during the current study are available from the corresponding author on reasonable request.

## Abbreviations

AHR: Adjusted hazard ratio
CI: Confidence interval
DTCSH: Debre Tabor Comprehensive Specialized Hospital
PIVC: Peripheral intravenous cannula
